# Kounis Syndrome Induced by Protamine Sulfate

**DOI:** 10.7759/cureus.6972

**Published:** 2020-02-12

**Authors:** Mohammad Amro, Kanaan Mansoor, Ahmed Amro, Kelechukwu Okoro, Paul I Okhumale

**Affiliations:** 1 Internal Medicine, Misr University for Science and Technology, Cairo, EGY; 2 Internal Medicine, Marshall University Joan C. Edwards School of Medicine, Huntington, USA; 3 Cardiology, Marshall University, Huntington, USA; 4 Cardiology, Marshall University Joan C. Edwards School of Medicine, Huntington, USA

**Keywords:** kounis, protamine, hypersensitivity

## Abstract

Protamine sulfate is considered a “life-saving” antidote for heparinized patients with major bleeds. Although the beneficial attributes and application of protamine sulfate in various clinical settings cannot be argued, it also has an impressive side-effect profile. Kounis syndrome (KS) is an acute coronary syndrome in the setting of an allergic reaction, which can be induced by numerous allergens. Herein, we report a case of KS secondary to the use of protamine sulfate after cryoablation for atrial fibrillation.

## Introduction

Protamine has been used to facilitate rapid sheath removal while still in-lab after percutaneous coronary interventions and catheter ablation procedures for many years. It has been shown to expedite ambulation without increased rates of thrombosis or access site complications. It can cause hypersensitivity reactions, leading to hypotension, rash, pulmonary edema, and, very rarely, Kounis syndrome (KS). KS has an incidence of 7.9-19.4 per 100,000 [1]. There are multiple inciting allergens associated with this condition and new triggers are constantly being discovered for this syndrome [1-2]. Herein, we report a case of KS secondary to the use of protamine sulfate after cryoablation for atrial fibrillation.

## Case presentation

**Figure 1:**
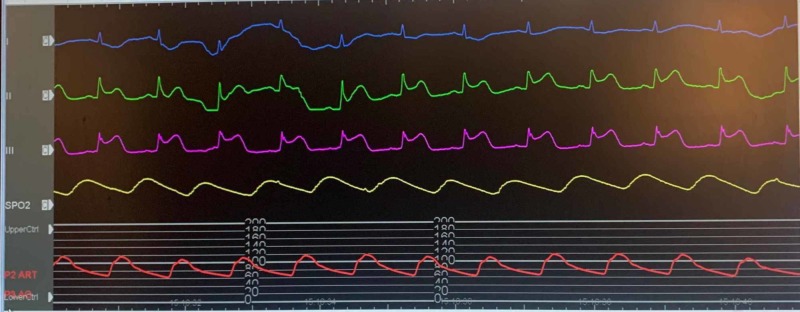
ST elevation in II and III

**Figure 2:**
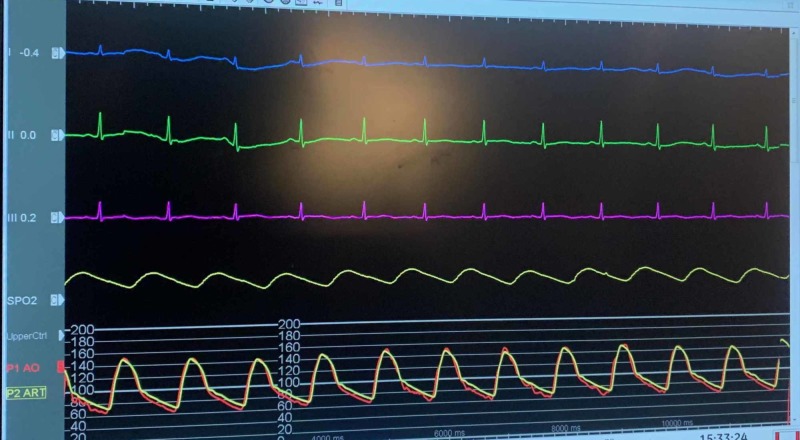
Post-cardiac catheterization, normalization of II and III

A 61-year-old Caucasian female with a pertinent past medical history of coronary artery disease status/post (s/p) coronary artery bypass grafting (CABG), diabetes mellitus, hypertension, and atrial fibrillation, who presented to the hospital for an elective cryoablation procedure of atrial fibrillation. The patient had successful cryoablation; post-procedure, protamine sulfate was administered to rapidly neutralize the effect of heparin to expedite sheath removal. While still on the table in the electrophysiology lab, the patient developed hypotension and the monitor showed ST elevations in the inferior leads, as shown in Figure 1. The patient underwent emergent coronary angiography while on the table in the same lab. Patient coronary angiography showed no acute findings. ST elevations and patient symptoms were short-lived for a few minutes and self-limiting and only required symptomatic treatment with intravenous fluids and supplemental oxygenation. Figure 2 shows a return of the ST segment to baseline in a few minutes while the patient is on the table and even before starting the catheterization. The rest of the hospital stay of the patient was uneventful and the patient was discharged home after protamine sulfate was listed as an allergy for her.

## Discussion

Protamine sulfate is considered a “life-saving” antidote for heparinized patients with major bleeds [3]. Although the beneficial attributes and application of protamine sulfate in various clinical settings cannot be argued, it also has an impressive side-effect profile [4]. In animal studies, it is associated with severe systemic hypotension, pulmonary artery hypertension, and respiratory distress [4]. These adverse effects are less pronounced in human beings but they still have a tendency to occur. Furthermore, protamine sulfate has also been associated with type 1 and type 3 immunological reactions and has the potential to instigate anaphylactoid reactions. Lindblad et al. in their review in 1989 reported that protamine sulfate is more likely to cause a type 1 anaphylactoid reaction, which occurs secondary to the release of histamine from mast cells [4]. The possible mechanism for a type 3 reaction to protamine sulfate occurs secondary to circulating immunoglobulin G (IgG) or anti-protamine immunoglobulin E (IgE) antibody, which are produced in response to prior exposure [4-6].

KS is believed to be caused by the vasospasm of coronary arteries secondary to the release of histamine and other vasoactive mediators from the degranulation of the mast cells [1,7-9]. Histamine plays an integral role in coronary hemodynamics and is stored within the coronary vasculature [1]. Histamine by virtue is a vasodilator but its effect on coronary arteries is concentration-dependent; it causes vasodilation at a low concentration via H1 receptors in the endothelium and H2 in smooth muscles while at a higher concentration, it causes vasoconstriction via the H1 receptors in the smooth muscles of the coronary arteries [10]. Other important mediators are proteases, namely, tryptase, chymase, cathepsin D, and thromboxane, disrupt the plaques, cause platelet aggregation and vasoconstriction, or increase angiotensin II, which causes more vasoconstriction in synergism with histamine. All these factors contribute to coronary vasospasm, leading to myocardial tissue damage [1]. KS is mainly a clinical diagnosis, confirmed by laboratory, electrocardiographic, echocardiographic, and angiographic evidence [1].

Hypersensitivity reactions may vary in intensity depending on the immunologic response of the patient. Given that protamine is an essential medication and is utilized post procedures after heparin use, one potential unproved approach could be premedication with anti-histamine, especially in patients with a shell-fish allergy. Though there is no association of shell allergy and the use of protamine sulfate, protamine is manufactured from salmon milt.

## Conclusions

Protamine sulfate administration leading to Kounis syndrome is very rare but reported in the literature. Physicians should be aware of this rare complication and be ready to manage it promptly.
